# Post-ERCP infection and its epidemiological and clinical characteristics in a large Chinese tertiary hospital: a 4-year surveillance study

**DOI:** 10.1186/s13756-017-0290-0

**Published:** 2017-12-29

**Authors:** Mingmei Du, Jijiang Suo, Bowei Liu, Yubin Xing, Liangan Chen, Yunxi Liu

**Affiliations:** 1 Department of Infection Management and Disease Control, Chinese PLA General Hospital, Fuxing Road No. 28, Beijing, 100853 China; 2 Department of Respiratory Medicine, Chinese PLA General Hospital, Fuxing Road No. 28, Beijing, 100853 China

**Keywords:** Endoscopic retrograde cholangiopancreatography, healthcare-acquired infections, epidemiology, cholangtitis, bacteremia, antimicrobial resistance, China

## Abstract

**Background:**

Endoscopic retrograde cholangiopancreatography (ERCP) is widely performed as a treatment for biliary and pancreatic illness in China; however, there are few data available regarding post-ERCP infections. This study aimed to describe the overall incidence of post-ERCP infections and the epidemiological characteristics of infected patients in a large tertiary-care hospital in China.

**Methods:**

Real-time surveillance was performed from 2012 through 2015 to identify all healthcare-associated infections (HAIs) that occurred after ERCP, using an automatic system. All HAIs (e.g., cholangtitis, bacteremia) were identified by infection control practitioners and doctors. Inpatient data were automatically collected by the surveillance system.

**Results:**

A total of 1743 ERCP operations were included in the study, among these, 132 (7.57%) HAIs were identified. ERCP postoperative infections occurred following different surgical procedures, with infection rates ranging from 3.58 to 13.51%. The most prevalent HAI was biliary tract infection (4.02%), followed by transient bacteremia (1.14%). Overall, 62 cases of bacteremia occurred following ERCP surgery and 34 (54.84%) cases occurred on the day of the operation or 1-day post-surgery. The most prevalent isolates detected during bacteremia were *Enterococcus faecium* (12/58) and *Escherichia coli* (11/58). A large proportion (72.73%) of the *E. coli* isolates and all of the *E. faecium* isolates were resistant to ciprofloxacin. In addition, only 37.50% of the *E. coli* isolates were susceptible to ceftriaxone.

**Conclusions:**

The high incidence of post-ERCP infection and the prevalence of drug resistance suggests that employing second generation cephalosporin or ceftriaxone as the antibiotic of choice for prophylaxis before ERCP, as recommended by the Chinese clinical application of antibacterial drugs guidelines, may not be effective.

## Background

China has a large number of patients with biliary or pancreatic diseases, especially bile duct stones. Currently, endoscopic retrograde cholangiopancreatography (ERCP) plays a major role in the treatment of biliary and pancreatic diseases. The use of ERCP has increased considerably in China in recent years. From 2006 to 2012, the total of ERCP surgeries performed increased from 63,787 to 195,643 in China, of which more than 95% were therapeutic [1]. Post-ERCP infections have been studied extensively in the USA and European countries, for example, surveillance of post-ERCP bacteremia showed infection rates of 5% in England [2] and 27.8% in the USA [3]. However, another retrospective observational study carried out in the USA over an 11-year period indicated that the post-ERCP infection rate was as low as 0.28% [4].

The variations in surveillance results for post-ERCP infections mainly result from different case definitions, varied surveillance durations and the different data collection methods used in previous studies [5]. To date, few studies have been conducted in China. One multicenter cross-sectional questionnaire study that aimed to determine the status of ERCP services in China, reported a cholangitis prevalence of 0.66% post-ERCP [1]. More research on understanding the incidence of adverse events following ERCP services is required in China.

This study aimed to describe the overall incidence of post-ERCP infection and characterize the infected patients after different ERCP operations. In addition, to improve our knowledge regarding the risk factors for post-ERCP infections and the effectiveness of prevention and treatment methods, our work determined the type of ERCP surgery associated with the highest incidence of healthcare-associated infections (HAI), and investigated the pathogenic spectrum and antibiotic resistance of the infecting bacteria. Our study involved surveillance in one of the largest tertiary hospitals in China from 2012 to 2015.

## Methods

### Definitions

1. Post-ERCP infection was defined as the HAI occurring on the day of operation or within 30 days post-surgery. The HAI criterion was consistent with that set by the Centers for Disease Control-National Healthcare Safety Network (CDC-NHSN) in the USA [6]. Infections observed for 30 days post-ERCP adhered to the Chinese criteria for post-surgery infections [7]. Infections definitely not caused by other operations unrelated to ERCP, within 30 days post-ERCP, were included.
2. Cholangitis was defined based on the Tokyo Guidelines 2007 [8] and the criteria included: 1) the presence of the following signs or symptoms after ERCP: fever (>38.0 °C), chill, abdominal pain, jaundice and liver biochemistry suggestive of biliary obstruction; 2) laboratory data indicative of the presence of inflammation and biliary obstruction; 3) imaging findings indicative of biliary obstruction, and 4) no evidence of acute cholangtitis in the week prior to the ERCP operation.
3. Bacteremia was defined as the presence of fever, chill and positive bacteria cultures from blood samples (excluding contamination, as previously described [9]) post-surgery, according to the definition of bloodstream infections from the CDC-NHSN [10].
  3.1-I) Secondary bacteremia was considered when the infection could definitely be sourced to one infected organ or site. For all cases of secondary bacteremia, the infection site was not recounted.
  3.1-II) Secondary bacteremia following an infection at another site/not related to ERCP was excluded.
  3.2-I) Transient primary bacteremia was counted at the infection site if positive bloodstream infections indicators (i.e., fever (>38.0 °C), chill) and positive blood culture results were observed.
  3.2-II) Transient primary bacteremia was counted at the infection site only if positive bloodstream infections indicators (i.e., fever (>38.0 °C), chill), confirmed the clinical diagnosis, and negative blood culture results were observed.

### Post-ERCP infection data collection

Post-ERCP infection data were collected at a tertiary hospital in Beijing, from 2012 to 2015. The hospital contained approximately 3800 beds, with 160 beds in the gastroenterology department. Each day, around two to four ERCP operations were performed in this hospital. Three senior clinical doctors, with more than 3 years of experience in ERCP operations, were in turn in charge of ERCP operations.

Real-time automatic hospital-wide surveillance of HAIs and outbreaks has been established in this hospital [11]. In brief, this system can automatically download microbiological reports, antibiotic usage, imaging reports, fever history and other information, and subsequently identify new HAIs in real time, and record and analyze the data. We used this system to collect post-ERCP infection data, including continuous fever (temperature ≥ 38.0 °C for >2 days), positive microbiological cultures and new antibiotic administration post-surgery. The system can develop post-ERCP HAI prewarning alerts. Subsequently, infection control practitioners work with doctors to confirm post-ERCP HAIs, as described in a previous study [11].

### Statistical analysis

Differences in categorical variables were assessed using a Pearson χ [2] test or Fisher’s exact test (when expected cell frequencies were <5). SPSS version 20.0 was used for all statistical analyses. A two-tailed *p* value of <0.05 was considered to be statistically significant.

## Results

### ERCP inpatients

From 2012 to 2015, a total of 1743 ERCP operations on 1660 patients were successfully carried out in the hospital. Of these cases, 1034 (62.29%) were male and 626 (37.71%) were female. Patients aged from 10 to 98 years old, with a median age of 61 years old. The median length of stay was 6 days. Reasons for ERCP included bile duct calculi (452, 27.23%), biliary pancreatic malignant tumors (365, 21.99%), obstructive jaundice (313, 18.86%), acute pancreatitis (224, 13.49%), cholangitis (157, 9.46%), cholelithiasis or cholecystitis (78, 4.70%) and other (71, 4.28%).

### Post-ERCP infection

Over the study period, a total of 132 HAIs occurred in 125 patients undergoing 1743 ERCP operations. The HAI prevalence was 7.57% (*n* = 132/1743). The median time to develop a post-ERCP infection was 7 days. Seven patients died and the other 1653 patients were discharged.

ERCP postoperative infections occurred following different surgical procedures, with infection rates ranging from 3.58 to 13.51%. The total number of HAIs and biliary tract infections that occurred following different types of operations are shown in Table 1. The three most prevalent post-ERCP infections were associated with bile duct or biliary stent implantation (13.51%), bile duct stent removal and replacement (10.42%) and bile duct stone removal operations (10.14%). There were no differences between the rates of infection for therapeutic ERCP (7.83%) and diagnostic ERCP (4.51%) (*p* = 0.165). The most prevalent HAI post-ERCP infections were biliary tract infections (4.02%), followed by transient bacteremia (1.14%), lower respiratory tract infections (0.91%), upper respiratory tract infections (0.54%), gastrointestinal infections (0.42%), urinary tract infections (0.18%) and others (0.42%).

**Table 1:** Prevalence of post-ERCP HAIs and biliary tract infections for different types of ERCP operations

Operations	No. of operations	No. (%) of HAIs	No. (%) of biliary tract infections
Diagnostic ERCP	133	6 (4.51)	5 (3.76)
Diagnostic ERCP	133	6 (4.51)	5 (3.76)
Therapeutic ERCP	1610	126 (7.83)	65 (4.03)
Lithotomy of duodenal papilla	727	26 (3.58)	7 (0.96)
Biliary stent implantation	570	77 (13.51)	47 (8.25)
Pancreatic duct stent implantation	137	7 (5.11)	0 (0)
Bile duct lithotomy	69	7 (10.14)	4 (5.80)
Bile duct stent extraction and replacement	48	5 (10.42)	3 (6.25)
Other therapeutic ERCP	59	4 (6.78)	4 (6.78)
Total	1743	132 (7.57)	70 (4.02)

HAIs included biliary tract infections, transient primary bacteremia, and respiratory tract infections

*ERCP* endoscopic retrograde cholangiopancreatography, *HAIs* healthcare-associated infections

### Post-ERCP bacteremia

A total of 62 bacteremia cases occurred post-ERCP, including 20 cases of transient and 42 cases of secondary bacteremia. All cases of bacteremia occurred within 14 days post-surgery. The majority of cases occurred on the day of surgery or 1 day post-surgery, which together accounted for 54.84% (34/62) of cases. All 20 cases of transient bacteremia occurred on the day of ERCP surgery and 1 day post-surgery. There were 42 cases of secondary bacteremia related to ERCP, including 39 secondary to biliary tract infections, one secondary to catheter-related bloodstream infections, one secondary to lower respiratory tract infections and one secondary to abdominal infections.

In addition, 58 pathogenic bacteria were cultivated from 62 patients. The majority (60.34%) were gram negative bacteria (Table 2). The most prevalent isolates were 12 *Enterococcus faecium* and 13 *Escherichia coli*. Overall, 72.73% of *E. coli* and 100.00% of *E. faecium* isolates were resistant to ciprofloxacin. In addition, only 37.50% of the *E. coli* isolates were susceptible to ceftriaxone. Gram-negative *E. coli* isolates were 100.00% susceptible to imipenem. The imipenem resistance rate for non-fermenting bacteria (five *Pseudomonas* and four *Acinetobacter baumannii*) reached 80.00–100.00%. Gram-positive *E. faecium* was 100.00% susceptible to vancomycin and linezolid (Table 3).

**Table 2:** The bacteriology of positive blood cultures post-ERCP

Organisms	No. (%) of organisms
Gram-positive organisms	22/58 (37.93%)
*Enterococcus faecium*	12/58 (20.69%)
*Enterococcus faecalis*	1/58 (1.72%)
*Staphylococcus spec.*	4/58 (6.90%)
Others	5/58 (8.62%)
Gram-negative organisms	35/58 (60.34%)
*Escherichia coli*	11/58 (18.97%)
*Klebsiella pneumonia*	5/58 (8.62%)
*Pseudomonas aeruginosa*	5/58 (8.62%)
*Enterobacter cloacae*	4/58 (6.90%)
*Acinetobacter baumannii*	4/58 (6.90%)
*Stenotrophomonas maltophilia*	2/58 (3.45%)
*Aeromonas spec.*	2/58 (3.45%)
Others	2/58 (3.45%)
Fungi	1/58 (1.72%)
*Candida glabrata*	1/58 (1.72%)

**Table 3:** Antibiotic susceptibilities of different blood culture isolates

Antibiotics	*Enterococcus faecium*	*Escherichia coli*	*Klebsiella pneumonia*	*Pseudomonas aeruginosa*	*Acinetobacter baumannii*	*Enterobacter cloacae*
Aamikacin	–	100%(11/11)	100%(5/5)	40%(2/5)	–	100%(4/4)
Ampicillin	8%(1/12)	14%(1/7)	0%(0/4)	0%(0/5)	0%(0/4)	0%(0/2)
Aztreonam	–	38%(3/8)	100%(4/4)	50%(2/4)	0%(0/3)	50%(1/2)
Nitrofurantoin	33%(4/12)	63%(5/8)	50%(2/4)	0%(0/3)	0%(0/3)	50%(1/2)
Ciprofloxacin	0%(0/12)	27%(3/11)	80%(4/5)	60%(3/5)	0%(0/4)	100%(4/4)
Gentamicin	38%(3/8)	75%(6/8)	100%(4/4)	33%(1/3)	33%(1/3)	100%(2/2)
Cefazolin	–	0%(0/6)	0%(0/4)	0%(0/3)	0%(0/3)	0%(0/2)
Ceftazidime	–	45%(5/11)	80%(4/5)	60%(3/5)	0%(0/4)	25%(1/4)
Ceftriaxone	–	38%(3/8)	75%(3/4)	0%(0/3)	0%(0/3)	50%(1/2)
Cefepime	–	64%(7/11)	80%(4/5)	40%(2/5)	0%(0/4)	75%(3/4)
Vancomycin	100%(12/12)	–	–	–	–	–
Linezolid	100%(12/12)	–	–	–	–	–
Imipenem	–	100%(11/11)	100%(5/5)	20%(1/5)	0%(0/4)	100%(4/4)
Levofloxacin	0%(0/8)	29%(2/7)	100%(4/4)	60%(3/5)	0%(0/3)	100%(2/2)

## Discussion

To our knowledge, this is the first report on post-ERCP infections in Chinese tertiary hospitals. Previous studies have indicated that therapeutic ERCP endoscopic procedures are associated with the highest rates of bacteremia and other infectious complications [3, 12]. Our study had a clear definition for HAIs post-ERCP operations, which excluded all infections that occurred before the ERCP. Using this definition, the overall prevalence of post-ERCP was 7.57%. However, it must be considered that, for example, if cholangitis occurred post-ERCP whether it was a complication of ERCP or just a natural history of gallbladder stones. The prevalence of bacteremia post-ERCP was 3.56% (62/1743) in our study, which was far lower than in other studies from other countries. Both Thosani et al. [3] in the USA and Kullman et al. [11] in Sweden reported the incidence of bacteremia associated with ERCP to be as high as 27%. The major reason for the high incidence might be that in these studies, two or three blood samples were obtained from each patient immediately before and after ERCP, to test the pathogenic spectrum. This active surveillance might increase the positive detection rate of bacteremia. In addition, these studies did not distinguish between primary and secondary bacteremia. Our study was based on daily medical work and only 17.73% (309/1743) of inpatients with suspected bacteremia provided samples for blood cultures.

In our study, the most prevalent infection post-ERCP was cholangitis (4.02%; 70/1743). Ertugrul et al. [13] reported a similar result, that 3.3% (17/503) of patients developed cholangitis after ERCP. In addition, the risk of cholangitis was significantly higher in patients with biliary dilatation and biliary stent insertion. Our study indicated that surgery for biliary tract disease was associated with the highest risk of infection. Surgery on the biliary tract frequently uses a basket or balloon lithotomy and biliary stent implantation is difficult and exposes patients to a high risk of bile duct injury. Bacterial colonization in the gallbladder or bile duct increases the risk of biliary tract infection post-surgery.

Our analysis showed the predominance of gram-negative organisms (60.34%) in the blood cultures, compared with gram-positive organisms (37.93%). The most prevalent isolates were *E. faecium* and *E. coli*. Rerknimitr et al. [14] reported similar findings in patients undergoing ERCP, which indicated that *Enterococcus spp.* and *E. coli* were the most prevalent organisms isolated from bile cultures. In addition, Thosani et al. [3] reported that *E. coli* and *Enterococcus spp.* were the most prevalent causes of bacteremia post-ERCP.

Park et al. [15] confirmed that *E. coli* and *Enterococcus spp.* were the most prevalent organisms isolated from the blood cultures of biliary tract-infected patients. According to patient outcomes, prophylactic antibiotics are thought to be the most effective prevention against infections from gram-negative bacteria and *Enterococcus spp*. Several guidelines [16–19] have been published about antibiotic prophylaxis prior to ERCP. Most guidelines [17, 18], such as the American Society for Gastrointestinal Endoscopy and the British Society of Gastroenterology Endoscopy, advocate prophylaxis for patients with biliary obstruction, whereas the European Society of Gastrointestinal Endoscopy [19] recommends prophylaxis for every type of therapeutic ERCP. Antibiotic prophylaxis involves administering an antibiotic to combat biliary microorganisms, such as *E. coli*, *Klebsiella pneumoniae* and *Enterococcus spp.* Oral ciprofloxacin, intravenous piperacillin-tazobactam or gentamicin is recommended in the USA [20].

In China, the excessive long-term use of antibiotics by hospitals and the widespread addition of quinolones to animal feed has led to the emergence of significant levels of bacterial resistance to many antimicrobials [21, 22]. For example, bacteria of the *Enterobacteriaceae* (especially *E. coli*) are resistant to fluoroquinolones and Xiao et al. reported that about 65% of *E. coli* strains were resistant to fluoroquinolones between 2004 and 2005 [21]. In our study, only 27% of *E. coli* isolates were found to be susceptible to ciprofloxacin. This limits the use of fluoroquinolones as prophylactic drugs in China. The 2015 Chinese Clinical Application of Antibacterial Drugs guidelines [23] recommended second generation cephalosporin or ceftriaxone as the antibiotics of choice for prophylaxis for the pathogenic bacteria that can cause skin incision infections after ERCP. However, all gram-negative organisms in our study were resistant to cefazolin. Nearly 90% of inpatients were treated with metronidazole and ceftriaxone prophylactic antibiotics in the study hospital; however, the drug susceptibility results revealed that only 38% of *E. coli* isolates were susceptible to ceftriaxone. The high infection rate after ERCP and the prevalence of drug-resistant pathogens indicate that further studies are important. A cost benefit analysis is also necessary to determine whether the recommended guidelines should be modified.

This study has several limitations. The rate of bacteremia may have been underestimated, because Chinese doctors in general less frequently submit blood specimens for bacterial culture [24]. Some cases of bacteremia would have therefore gone undetected because of a lack of blood culture results. Another issue is that our study monitored all ERCP cases during hospitalization but post-discharge surveillance was not carried out at 30 days post-ERCP procedure. Post-discharge surveillance could be performed to identify HAIs that were not been reported and documented in our research.

## Conclusions

Compared with HAIs in the whole hospital [24], post-ERCP infections generally exhibited a higher incidence (7.6% vs. 4.1%) and different types of infections (cholangitis vs. respiratory tract infections). The detailed epidemiological data for HAIs after ERCP in China may improve our understanding of the severity of post-ERCP infections and potential prevention and treatment measures. Our study indicates that the incidence of post-ERCP infection is high and that drug resistance among the causative bacteria is common; thus, we suggest that the use of second generation cephalosporin or ceftriaxone as the antibiotic of choice for prophylaxis before ERCP may not be effective. Therefore, we recommend administering a high-grade antibiotic (i.e. piperacillin-tazobactam), which would target most biliary organisms, before ERCP.

## Abbreviations

CDC-NHSN: Centers for Disease Control-National Healthcare Safety Network
ERCP: Endoscopic retrograde cholangiopancreatography
HAI: Healthcare-associated infection
